# Supplementary material for: Association between INDELs in MicroRNAs and Susceptibility to Gastric Cancer in Amazonian Population
Source: Genes (Basel). 2022 Dec 24;14(1):60. doi: 10.3390/genes14010060 (PMC9858651; doi:10.3390/genes14010060)
Supplement: Supplementary file 1 [file genes-14-00060-s001.zip › genes-1964826-supplementary.pdf]

SUPLEMENTARY MATERIALS

Table S1. Genotypic and allelic frequency of the 11 INDELs regarding susceptibility to GC. Data presented as percentage of patients with GC/percentage of controls. dbSNP: Genetic variant record in the NCBI database. HWE: Hardy–Weinberg Equilibrium.

Gene	dbSNP	Case/control (*n* = *301*/145)						
		Genotype frequency				Allele frequency		HWE
		II	ID	DD	*P* value	I	D	*P* value
MIRNA630	rs139334001	89,7/92,4	9,0/7,6	1,3/0,0	0,440	94,8	5,16	0,023
MIRNA516B_2	rs10670323	1,0/2,1	19,0/19,4	80/78,5	0,66	10,5	89,5	0,631
MIRNA4463	rs5877455	28,6/41,6	49,8/43,1	21,5/15,3	0,023	56,5	43,4	0,558
MIRNA3171	rs35170395	69,2/75	24/25	6,8/0	0,001	83,2	16,74	0,009
MIRNA3945	rs145931056	80,3/88,8	18,4/9,8	1,4/1,4	0,055	90,8	9,15	0,239
MIRNA548H_4	rs150141473	74,8/83,9	24,1/15,4	1,0/0,7	0,090	88,4	11,5	0,489
MIRNA548AJ_2	rs145326096	67,2/75,9	9,6/7,6	23,2/16,6	0,166	74,5	25,4	0,000
MIRNA302C	rs199971565	96,6/100	2/0,0	1,4/0,0	0,076	98,4	1,59	0,000
MIRNA4274	rs202195689	90,9/85,2	8,4/14,8	0,7/0,0	0,092	94,2	5,7	0,642
MIRNA920	rs66686007	91,5/82,1	8,2/16,6	0,3/1,4	0,015	93,8	6,15	0,217
MIRNA3652	rs62747560	62,2/62,7	35,0/27,5	2,7/9,9	0,004	78,6	21,3	0,567

Table S2. Allelic variants in MIRNAs associations for GC predisposition. Logistic regression analysis with Bonferroni correction. Genotypes: Del / Del = homozygous deletion, Del / Ins = heterozygous, and Ins-Ins = homozygous insertion.

Gene	Model	OR (IC 95%)	*P* value
MIRNA630_rs139334001	Ins / Ins vs Del / Ins + Del / Del	1,40 (0,68-2,88)	0,344
	Ins / Ins + Del / Ins vs Del / Del	1,00 (0,68-2,0)	0,308
MIRNA516B_2_rs10670323	Ins / Ins vs Del / Ins + Del / Del	1,00 (0,50-1,50)	0,37
	Ins / Ins + Del / Ins vs Del / Del	0,91 (0,56-1,48)	0,709
*MIRNA4463_rs5877455_*	Ins / Ins vs Del / Ins + Del / Del	1,78 (1,16-2,71)	0,007
	Ins / Ins + Del / Ins vs Del / Del	1,00 (0,65-1,65)	0,122
MIRNA3171_rs35170395	Ins / Ins vs Del / Ins + Del / Del	1,34 (0,85-2,10)	0,203
	Ins / Ins + Del / Ins vs Del / Del	1,00 (0,42-1,50)	0
MIRNA3945_rs145931056	Ins / Ins vs Del / Ins + Del / Del	1,95 (1,08-3,53)	0,021
	Ins / Ins + Del / Ins vs Del / Del	1,00 (0,18-5,37)	0,974
MIRNA548H_4_rs150141473	Ins / Ins vs Del / Ins + Del / Del	1,75 (1,05-2,95)	0,028
	Ins / Ins + Del / Ins vs Del / Del	1,00 (0,15-14,2)	0,734
MIR548AJ_2_rs145326096	Ins / Ins vs Del / Ins + Del / Del	1,53 (0,97-2,41)	0,06
	Ins / Ins + Del / Ins vs Del / Del	1,00 (0,91-2,55)	0,102
MIRNA302C_rs199971565	Ins / Ins vs Del / Ins + Del / Del	1,00 (0,30-1,80)	0,034
	Ins / Ins + Del / Ins vs Del / Del	1,00 (0,35-2,00)	0,307
MIRNA4274_rs202195689	Ins / Ins vs Del / Ins + Del / Del	0,58 (0,31-1,06)	0,081
	Ins / Ins + Del / Ins vs Del / Del	1,00 (0,80-2,00)	1
MIRNA920_rs66686007	Ins / Ins vs Del / Ins + Del / Del	0,43 (0,24-0,77)	0,004
	Ins / Ins + Del / Ins vs Del / Del	0,24 (0,02-2,71)	0,232
MIRNA3652_rs62747560	Ins / Ins + Ins / Del vs Del / Del	0,26 (0,10-0,62)	0,002
	Ins / Ins vs Del / Ins + Del / Del	1,02 (0,67-1,54)	0,93

Table S3. Significant associations of insertions and deletions in the analysis of the 11 INDELs regarding clinical variants in the GC. Genotypes: Del / Del = homozygous deletion, Del / Ins = heterozygous, and Ins-Ins = homozygous insertion.

			OR (IC 95%)	*P* value
MIRNA630_rs139334001	Ins / Ins vs Del / Ins + Del / Del	Lauren (Diffuse or Intestinal)	1,01 (0,46-2,21)	0,975
	Ins / Ins + Del / Ins vs Del / Del		1,00 (0,20-10,38)	0,718
MIRNA516B_2_rs10670323	Ins / Ins vs Del / Ins + Del / Del		1,00 (0,30-2,10)	0,514
	Ins / Ins + Del / Ins vs Del / Del		1,43 (0,79-2,61)	0,242
*MIRNA4463_rs5877455_*	Ins / Ins vs Del / Ins + Del / Del		2,30 (1,27-4,18)	0,004
	Ins / Ins + Del / Ins vs Del / Del		1,00 (0,83-2,22)	0,216
MIRNA3171_rs35170395	Ins / Ins vs Del / Ins + Del / Del		0,88 (0,51-1,50)	0,636
	Ins / Ins + Del / Ins vs Del / Del		1,00 (0,21-1,78)	0,351
MIRNA3945_rs145931056	Ins / Ins vs Del / Ins + Del / Del		1,22 (0,65-2,27)	0,539
	Ins / Ins + Del / Ins vs Del / Del		1,00 (0,05-4,79)	0,52
MIRNA548H_4_rs150141473	Ins / Ins vs Del / Ins + Del / Del		1,46 (0,84-2,53)	0,179
	Ins / Ins + Del / Ins vs Del / Del		1,00 (0,07-8,43)	0,816
MIR548AJ_2_rs145326096	Ins / Ins vs Del / Ins + Del / Del		1,41 (0,84-2,37)	0,199
	Ins / Ins + Del / Ins vs Del / Del		1,00 (0,47-1,53)	0,574
MIRNA302C_rs199971565	Ins / Ins vs Del / Ins + Del / Del		1,92 (0,50-7,31)	0,338
	Ins / Ins + Del / Ins vs Del / Del		1,00 (0,05-4,83)	0,524
MIRNA4274_rs202195689	Ins / Ins vs Del / Ins + Del / Del		1,39 (0,57-3,39)	0,474
	Ins / Ins + Del / Ins vs Del / Del		1,00 (0,10-0,95)	0,402
MIRNA920_rs66686007	Ins / Ins vs Del / Ins		1,00 (0,19-1,62)	0,271
MIRNA3652_rs62747560	Ins / Ins vs Ins / Del + Del / Del		0,97 (0,59-1,62)	0,922
	Ins / Ins + Del / Ins vs Del / Del		1,00 (0,56-17,29)	0,178
MIRNA630_rs139334001	Ins / Ins vs Del / Ins + Del / Del	Location (Cardia or non-cardia)	0,83 (0,37-1,88)	0,659
	Ins / Ins + Del / Ins vs Del / Del		1,00 (0,20-10,38)	0,01
MIRNA516B_2_rs10670323	Ins / Ins vs Del / Ins + Del / Del		1,00 (0,03-7,57)	0,596
	Ins / Ins + Del / Ins vs Del / Del		1,22 (0,64-2,33)	0,534
*MIRNA4463_rs5877455_*	Ins / Ins vs Del / Ins + Del / Del		2,20 (1,26-3,84)	0,005
	Ins / Ins + Del / Ins vs Del / Del		1,00 (0,71-2,57)	0,344
MIRNA3171_rs35170395	Ins / Ins vs Del / Ins + Del / Del		0,81 (0,47-1,42)	0,469
	Ins / Ins + Del / Ins vs Del / Del		1,00 (0,21-1,47)	0,246
MIRNA3945_rs145931056	Ins / Ins vs Del / Ins + Del / Del		0,78 (0,41-1,46)	0,435
	Ins / Ins + Del / Ins vs Del / Del		1,00 (0,02-1,48)	0,07
MIRNA548H_4_rs150141473	Ins / Ins vs Del / Ins + Del / Del		0,90 (0,50-1,61)	0,72
	Ins / Ins + Del / Ins vs Del / Del		1,00 (0,02-2,54)	0,21
MIR548AJ_2_rs145326096	Ins / Ins vs Del / Ins + Del / Del		2,01 (1,11-3,63)	0,017
	Ins / Ins + Del / Ins vs Del / Del		1,00 (0,66-2,33)	0,502
MIRNA302C_rs199971565	Ins / Ins vs Del / Ins + Del / Del		3,86 (0,48-31,39)	0,137
	Ins / Ins + Del / Ins vs Del / Del		1,00 (0,14-13,74)	0,762
MIRNA4274_rs202195689	Ins / Ins vs Del / Ins + Del / Del		0,62 (0,26-1,46)	0,283
	Ins / Ins + Del / Ins vs Del / Del		1,00 (0,10-0,95)	1
MIRNA920_rs66686007	Ins / Ins vs Del / Ins		3,02 (0,86-10,54)	0,053
MIRNA3652_rs62747560	Ins / Ins vs Ins / Del + Del / Del		0,73 (0,43-1,23)	0,24
	Ins / Ins + Del / Ins vs Del / Del		1,00 (0,21-17,25)	0,545
MIRNA630_rs139334001	Ins / Ins vs Del / Ins + Del / Del		1,58 (0,74-3,39)	0,232

	Ins / Ins + Del / Ins vs Del / Del		1,00 (0,13-6,86)	0,961
MIRNA516B_2_rs10670323	Ins / Ins vs Del / Ins + Del / Del		1,00 (0,10-0,95)	0,237
	Ins / Ins + Del / Ins vs Del / Del		1,33. (0,75-2,38)	0,331
*MIRNA4463_rs5877455_*	Ins / Ins vs Del / Ins + Del / Del		2,80 (1,64-4,80)	0
	Ins / Ins + Del / Ins vs Del / Del		1,00 (0,19-1,62)	0
MIRNA3171_rs35170395	Ins / Ins vs Del / Ins + Del / Del		1,00 (0,01-0,06)	0
	Ins / Ins + Del / Ins vs Del / Del		0,12 (0,07-0,21)	0
MIRNA3945_rs145931056	Ins / Ins vs Del / Ins + Del / Del		1,34 (0,74-2,42)	0,334
	Ins / Ins + Del / Ins vs Del / Del	Early or late diagnosis (less o more than 40 years)	1,00 (0,07-8,43)	0,058
MIRNA548H_4_rs150141473	Ins / Ins vs Del / Ins + Del / Del		0,97 (0,57-1,66)	0,918
	Ins / Ins + Del / Ins vs Del / Del		1,00 (0,59-1,74)	0,969
MIR548AJ_2_rs145326096	Ins / Ins vs Del / Ins + Del / Del		1,00 (0,87-5,07)	0
	Ins / Ins + Del / Ins vs Del / Del		7,41 (3,34-16,44)	0
MIRNA302C_rs199971565	Del / Del vs Ins / Del		1,00 (0,14-7,15)	0,994
MIRNA4274_rs202195689	Ins / Ins vs Del / Ins + Del / Del		0,98 (0,44-2,19)	0,955
	Ins / Ins + Del / Ins vs Del / Del		1,00 (0,13-6,86)	0,498
MIRNA920_rs66686007	Ins / Ins vs Del / Ins		1,59 (0,67-3,80)	0,385
MIRNA3652_rs62747560	Ins / Ins vs Ins / Del + Del / Del		0,96 (0,59-1,55)	0,867
	Ins / Ins + Del / Ins vs Del / Del		1,00 (0,07-2,04)	0,239
MIRNA630_rs139334001	Ins / Ins vs Del / Ins + Del / Del		0,23 (0,03-1,77)	0,08
	Ins / Ins + Del / Ins vs Del / Del		1,00 (0,02-2,54)	1
MIRNA516B_2_rs10670323	Ins / Ins vs Del / Ins + Del / Del		1,00 (0,45-121,44)	0,19
	Ins / Ins + Del / Ins vs Del / Del		0,98 (0,38-2,52)	0,96
*MIRNA4463_rs5877455_*	Ins / Ins vs Del / Ins + Del / Del		0,52 (0,23-1,13)	0,105
	Ins / Ins + Del / Ins vs Del / Del		1,00 (0,16-1,39)	0,141
MIRNA3171_rs35170395	Ins / Ins vs Del / Ins + Del / Del		0,65 (0,27-1,58)	0,326
	Ins / Ins + Del / Ins vs Del / Del		1,00 (0,19-3,94)	0,845
MIRNA3945_rs145931056	Ins / Ins vs Del / Ins + Del / Del		0,59 (0,19-1,76)	0,315
	Ins / Ins + Del / Ins vs Del / Del	Tumor staging T1 vs T2 / T3 / T4	1,00 (0,24-23,71)	0,183
MIRNA548H_4_rs150141473	Ins / Ins vs Del / Ins + Del / Del		0,75 (0,31-1,83)	0,52
	Ins / Ins + Del / Ins vs Del / Del		1,00 (0,32-40,92)	0,344
MIR548AJ_2_rs145326096	Ins / Ins vs Del / Ins + Del / Del		0,62 (0,25-1,50)	0,27
	Ins / Ins + Del / Ins vs Del / Del		1,00 (0,30-1,97)	0,572
MIRNA302C_rs199971565	Ins / Ins vs Del / Ins + Del / Del		0,00 (0,00 -0,00)	0,606
	Ins / Ins + Del / Ins vs Del / Del		1,00 (0-0)	1
MIRNA4274_rs202195689	Ins / Ins vs Del / Ins		0,4 (0,05-3,09)	0,314
MIRNA920_rs66686007	Ins / Ins vs Del / Ins		0,42 (0,05-3,29)	0,352
MIRNA3652_rs62747560	Ins / Ins vs Ins / Del + Del / Del		1,48 (0,69-3,16)	0,316
	Ins / Ins + Del / Ins vs Del / Del		1,00 (0,02-2,54)	1
